# Supplementary material for: Large transient assemblies of Apaf1 constitute the apoptosome in cells
Source: Nat Commun. 2025 Oct 24;16:9429. doi: 10.1038/s41467-025-64478-9 (PMC12552632; doi:10.1038/s41467-025-64478-9)
Supplement: Supplementary file 1 — Supplementary Information [file 41467_2025_64478_MOESM1_ESM.pdf]

SUPPLEMENTARY INFORMATION

Large transient assemblies of Apaf1 constitute the apoptosome in cells

Alicia C. Borgeaud[1,2], Iva Ganeva[1,2], Calvin Klein[1,3], Amandine Stooss[1], Daniela Ross-Kaschitza[1], Liyang Wu[4], Joel S. Riley[5,6,7], Stephen W.G. Tait[5,6], Thomas Lemmin[1], Thomas Kaufmann[4], Wanda Kukulski[1,2*]

[1]Institute of Biochemistry and Molecular Medicine, University of Bern, 3012 Bern, Switzerland
[2]MRC Laboratory of Molecular Biology, Cambridge CB2 0QH, UK
[3]Graduate School for Cellular and Biomedical Sciences, University of Bern, 3012 Bern, Switzerland
[4]Institute of Pharmacology, University of Bern, 3010 Bern, Switzerland
[5]Cancer Research UK Scotland Institute, Glasgow G61 1BD, UK
[6]School of Cancer Sciences, College of Medical, Veterinary and Life Sciences, University of Glasgow, Glasgow G61 1QH, UK
[7]Institute of Developmental Immunology, Biocenter, Medical University of Innsbruck, 6020 Innsbruck, Austria
*Correspondence to: wanda.kukulski@unibe.ch

This Supplementary Information file includes:
Supplementary Figures 1 to 5
Supplementary Table 1
Supplementary references

SUPPLEMENTARY FIGURES

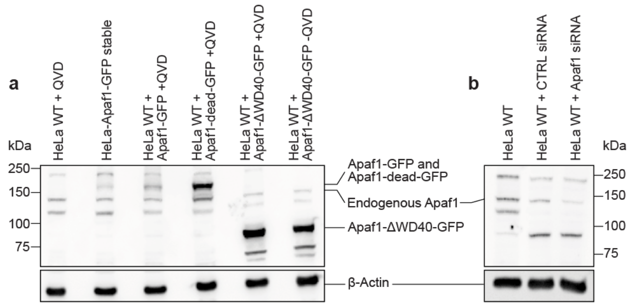

Supplementary Fig. 1: Apaf1 expression. a) Western blot of untransfected HeLa cells, HeLa cells stably or transiently expressing Apaf1-GFP (169 kDa), or transiently expressing Apaf1-dead-GFP (169 kDa) or Apaf1-ΔWD40-GFP (91 kDa) in presence or absence of QVD as indicated, detected using an anti-Apaf1 antibody. Endogenous Apaf1 is expected at 142 kDa. An antibody against β-actin was used for the loading control and detected using ECL. Representative of four experiments with similar results. **b)** Western blot of untransfected HeLa cells, HeLa cells expressing a control siRNA, and HeLa cells expressing an Apaf1 siRNA. The expression of endogenous Apaf1 decreases upon expression of the Apaf1 siRNA. An antibody against β-actin was used for the loading control and detected using fluorescence. Representative of two experiments with similar results.

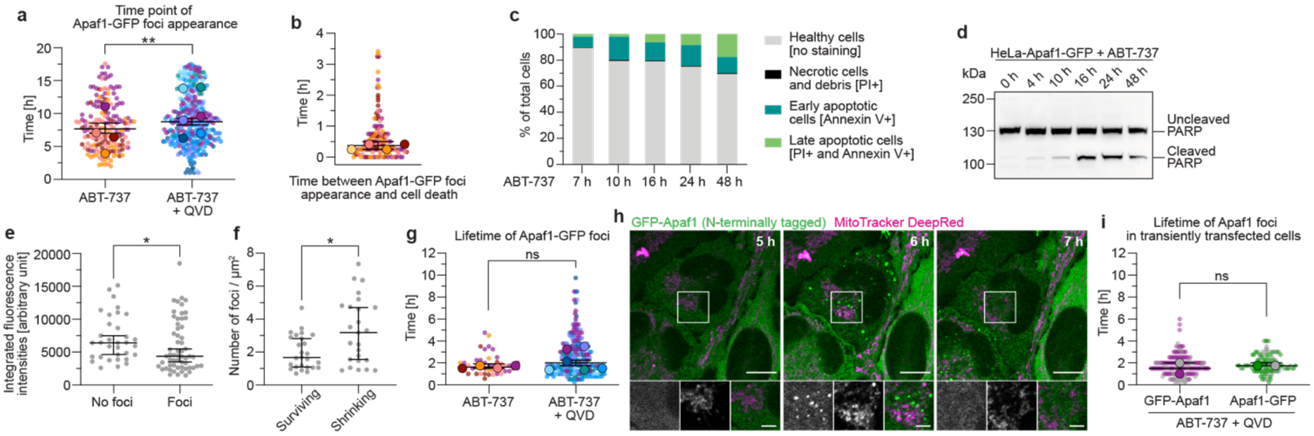

Supplementary Fig. 2: Characteristics of Apaf1 foci and cell death in HeLa cells. a) Time points of Apaf1-GFP foci appearance in HeLa cells stably expressing Apaf1-GFP, since treatment with ABT-737, in absence or presence of QVD. Each point represents an individual cell. Each colour represents an experiment[1]. Medians of each experiment are indicated by large points. Black lines indicate medians with 95% confidence interval of all data points combined. Treated with ABT-737: Median: 7 h 40 min, MAD: 2 h 35 min, N=173 cells examined over 4 independent experiments. Treated with ABT-737 and QVD: Median: 8 h 45 min, MAD: 2h 45 min, N=324 cells examined over 6 independent experiments. P-value=0.0054 (two-tailed Mann-Whitney test). **b)** Time elapsed between Apaf1-GFP foci appearance and cell shrinkage (indicating cell death) in HeLa cells stably expressing Apaf1-GFP, treated with ABT-737. Each point represents an individual cell. Each colour represents an experiment. Medians of each experiment are indicated by large points. Black lines indicate median with 95% confidence interval of all data points combined. Median: 23 min, MAD: 17 min, N=144 cells examined over 4 independent experiments. **c)** Percentages of healthy (grey), necrotic (black), early apoptotic (dark green), or late apoptotic (light green) HeLa cells stably expressing Apaf1-GFP at different time points after ABT-737 treatment, determined by FACS using propidium iodide (PI) and Annexin V staining. N=5000 cells examined for each condition over 1 experiment. **d)** Western blot showing poly(ADP-ribose) polymerase-1 (PARP) cleavage, indicative of effector caspase activity, in HeLa cells stably expressing Apaf1-GFP at different time points after ABT-737 treatment, detected using an anti-PARP antibody. Representative of 4 experiments with similar results. **e)** Integrated fluorescence intensities of HeLa cells stably expressing Apaf1-GFP, determined in a frame 5 minutes before shrinkage, indicating cell death (for cells without foci), or 5 minutes before formation of Apaf1 foci (for cells with foci). Dots represent individual cells. Black lines indicate medians with 95% confidence interval, in arbitrary units. Cells without foci: Median: 6415, MAD: 2249. N=33 cells. Cells with foci: Median: 4367, MAD: 1954, N=58 cells. P-value=0.0303 (two-tailed Mann-Whitney test). Images used are a subset of the data used in Figure 1. **f)** Number of Apaf1-GFP foci per μm^2 of cellular area in cells shrinking (indicating cell death) or surviving following Apaf1 foci formation. Dots represent individual cells. The cells analysed here are a subset of the cells analysed in Figure 1c, randomly selected. Black lines indicate medians with 95% confidence interval. Surviving: Median: 1.7 foci / μm^2, MAD: 0.7 foci / μm^2, N=26 cells. Shrinking: Median: 3.2 foci / μm^2, MAD 1.6 foci / μm^2, N=25 cells. P-value=0.0247 (two-tailed Mann-Whitney test). **g)** Lifetimes of Apaf1-GFP foci in HeLa cells stably expressing Apaf1-GFP treated with ABT-737, in presence or absence of QVD. Each dot represents an individual cell. Each colour represents an experiment. Medians of each experiment are indicated by large points. Black lines indicate median with 95% confidence interval of all data points combined. For ABT-737 treated cells: Median: 1 h 38 min, MAD: 23 min, N=44 cells examined over 4 independent experiments. For ABT-737 and QVD treated cells: Median: 2 h, MAD: 1 h, N=263 cells examined over 6 independent

experiments. P-value=0.0581 (two-tailed Mann-Whitney test). **h)** Live fluorescence imaging of HeLa cells transiently expressing a GFP-Apaf1 construct with the GFP-tag at the N-terminus, showing Apaf1 foci (green) upon ABT-737 and QVD treatment, similarly to the C-terminal constructs that were used throughout the study. Mitochondria were stained with MitoTracker DeepRed (magenta). Image acquisition time since ABT-737 and QVD treatment is indicated on large images. White squares indicate areas shown as close-ups (from left to right: GFP-Apaf1; MitoTracker DeepRed; merge). Scale bars: 10 µm in large images, 3 µm in close-ups. **i)** Lifetimes of Apaf1-GFP or GFP-Apaf1 foci in transiently transfected HeLa cells treated with ABT-737 and QVD. Each dot represents an individual cell. Each colour represents an experiment[1]. Medians of each experiment are indicated by large points. Black lines indicate median with 95% confidence interval of all data points combined. For GFP-Apaf1 transfected cells: Median: 1 h 30 min, MAD: 30 min, N=165 cells examined over 2 independent experiments. For Apaf1-GFP transfected cells: Mean: 1 h 45 min, MAD: 30 min, N=117 cells examined over 2 independent experiments. P-value=0.1774 (two-tailed Mann-Whitney test).

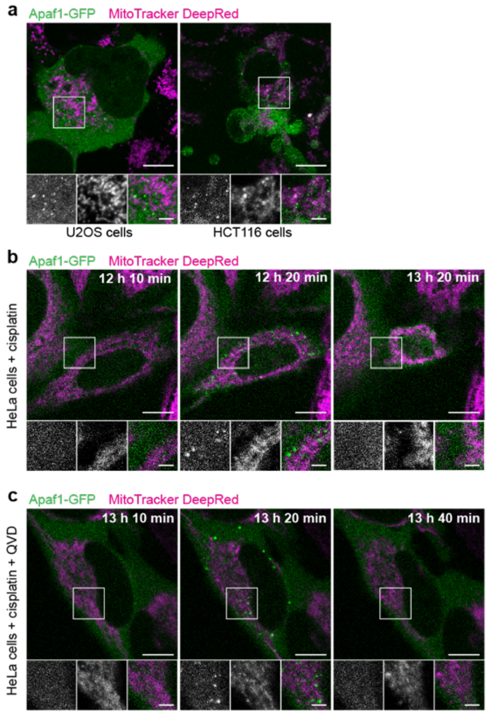

Supplementary Fig. 3: Apaf1 foci form also in U2OS and HCT116 cells and upon cisplatin treatment. a) Live fluorescence imaging of U2OS and HCT116 cells, transiently expressing Apaf1-GFP (green), showing Apaf1 foci formation upon ABT-737 treatment. Mitochondria were stained with MitoTracker DeepRed (magenta). White squares indicate areas shown as close-ups (from left to right: Apaf1-GFP; MitoTracker DeepRed; merge). Images are representative of three experiments each. **b)** Live fluorescence imaging of HeLa cells stably expressing Apaf1-GFP, showing Apaf1 foci (green) upon cisplatin treatment. Mitochondria were stained with MitoTracker DeepRed (magenta). Image acquisition time since ABT-737 treatment is indicated on large images. White squares indicate areas shown as close-ups (from left to right: Apaf1-GFP; MitoTracker DeepRed; merge). **c)** Live fluorescence imaging of HeLa cells stably expressing Apaf1-GFP, showing Apaf1 foci (green) upon cisplatin and QVD treatment. Mitochondria were stained with MitoTracker DeepRed (magenta). Image acquisition time since ABT-737 treatment is indicated on large images. White squares indicate areas shown as close-ups (from left to right: Apaf1-GFP; MitoTracker DeepRed; merge). Images in b and c are representative of two experiments. Scale bars in a, b and c: 10 µm in large images, 3 µm in close-ups.

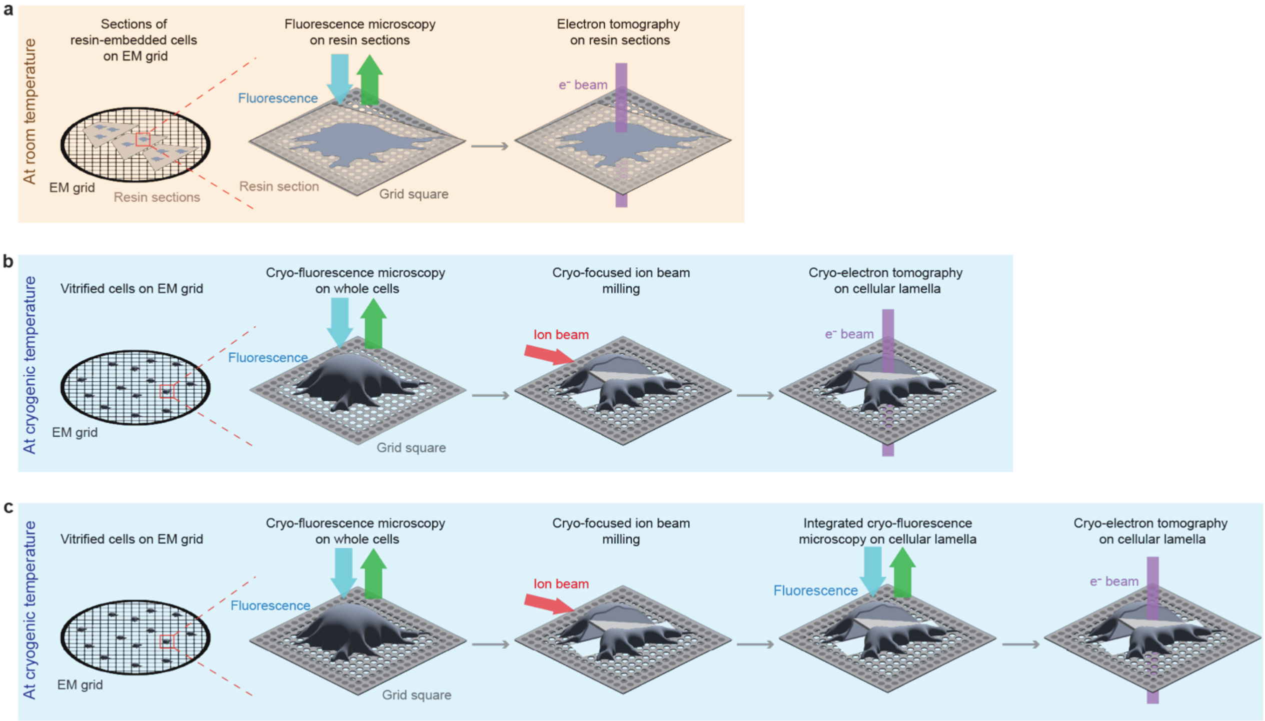

Supplementary Fig. 4: Correlative light and electron microscopy (CLEM) workflows to identify and image the structural organization of Apaf1 foci in cells. a) In-resin CLEM[2, 3]: After high-pressure freezing, freeze-substitution and embedding, samples are sectioned into approximately 200 nm thin sections, which are deposited on electron microscopy (EM) grids. The grids are imaged by fluorescence microscopy to identify regions of the sections containing cell areas with fluorescent signals of interest, here Apaf1-GFP foci. These areas are found back in the electron microscope, and electron tomograms are acquired at room temperature. The position of the tomograms is correlated with high precision to the fluorescence images to determine the centroid positions of the Apaf1-GFP foci. **b)** Pre-focused ion beam (FIB) milling cryo-CLEM[3, 4]: Cells are grown on EM grids and vitrified by plunge freezing before the EM grids are imaged by cryo-fluorescence microscopy to identify cells containing fluorescent signals of interest, here Apaf1-GFP foci. The EM grids are then transferred to a cryo-FIB scanning electron microscope (SEM), where cells identified to contain Apaf1-GFP foci are thinned to obtain approximately 200 nm thin lamella. The EM grids containing lamella are then transferred into a cryo-transmission electron microscope (TEM) for cryo-electron tomography (cryo-ET). The regions of cryo-ET acquisition are selected based on correlation of the whole-cell cryo-fluorescence images with lamellae overview images. This approach is less precise than the correlation obtained through the workflows shown in a and c. **c)** Pre- and post-FIB milling cryo-CLEM[5, 6]: Cells are grown on EM grids and vitrified by plunge freezing before the EM grids are imaged by cryo-fluorescence microscopy to identify cells containing the fluorescent signals of interest, here Apaf1-SNAP647 foci. The EM grids are then transferred to a cryo-FIB SEM, where cells identified to contain signals of Apaf1-SNAP647 foci are thinned to obtain approximately 200 nm thin lamella. To confirm presence and precise location of the signals of interest in the lamellae post-milling, the lamellae are imaged using an integrated cryo-fluorescence microscope within the cryo-FIB-SEM[7]. The EM grids are then transferred into a cryo-TEM for cryo-ET. The regions of cryo-ET acquisition are selected based on correlation of post-FIB milling cryo-fluorescence images with lamellae overview cryo-TEM images, allowing a precise localisation of Apaf1-SNAP647 foci signals.

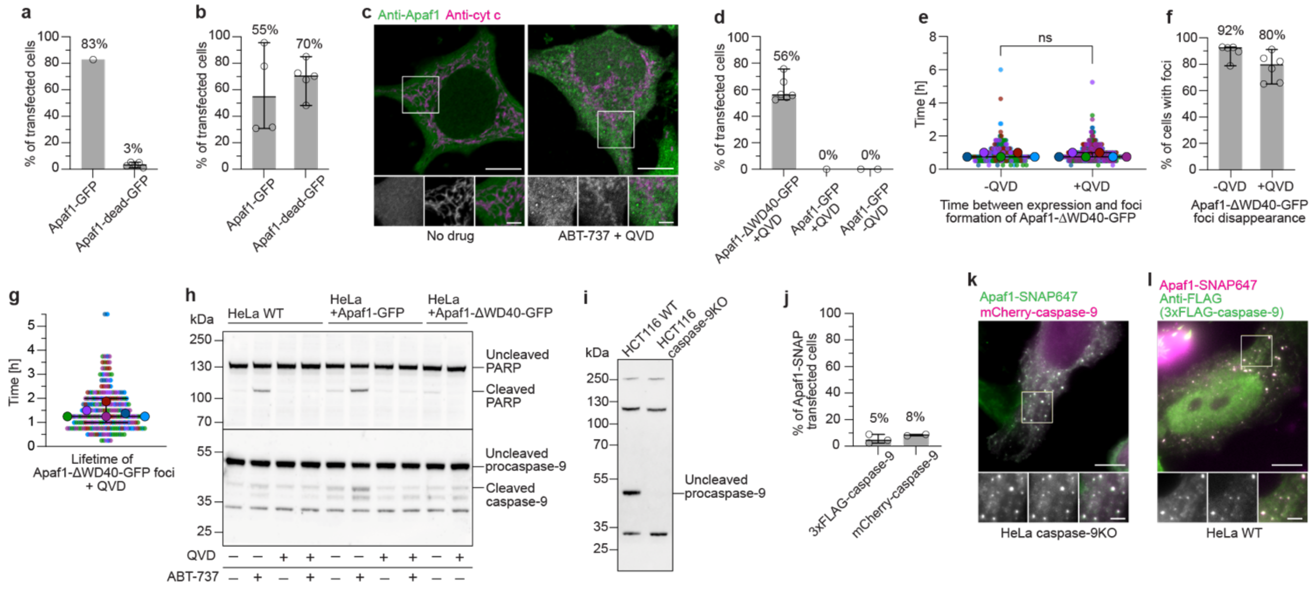

Supplementary Fig. 5: Apaf1-dead-GFP and Apaf1-ΔWD40-GFP foci formation and characteristics in HeLa cells, as well as caspase-9 localisation. a) Percentages of HeLa cells transiently transfected with Apaf1-GFP or Apaf1-dead-GFP, treated with ABT-737 and QVD, showing Apaf1 foci formation, among all transfected cells. Note that a similar experiment is shown in Figure 5c but without QVD. Lines correspond to median with 95% confidence interval. Points correspond to percentage per experiment. Apaf1-GFP: 83%, N= 76 cells examined over 1 experiment. Apaf1-dead-GFP: Median: 3%, MAD: 2%, N= 450 cells examined over 5 independent experiments. At least 60 cells were imaged per condition and experiment. **b)** Percentages of ABT-737-treated HeLa cells transiently expressing Apaf1-GFP or Apaf1-dead-GFP that shrank, indicating cell death, among all transfected cells. Lines correspond to medians with 95% confidence interval. Apaf1-GFP: Median: 55%, MAD: 23%, N=592 cells examined over 4 independent experiments. Apaf1-dead-GFP: Median: 70%, MAD: 3%, N=446 cells examined over 5 independent experiments. At least 66 cells were imaged per condition and experiment. **c)** Immunofluorescence of untreated and ABT-737/QVD-treated HeLa cells, using antibodies labelling endogenous Apaf1 (green) and cyt c (magenta). White squares indicate areas shown as close-ups (from left to right: Apaf1; cyt c; merge). Images are representative of 3 experiments with similar results. **d)** Percentages of HeLa cells transiently transfected with Apaf1-ΔWD40-GFP or Apaf1-GFP forming foci without induction of apoptosis by ABT-737, in presence or absence of QVD as indicated, among all transfected cells. Note that a similar experiment is shown in Figure 5f but without QVD. Black lines correspond to medians with 95% confidence interval. Apaf1-ΔWD40-GFP: Median: 56%, MAD: 3%, N=561 cells examined over 6 independent experiments. Apaf1-GFP with QVD: 0% N=91 cells examined over 1 experiment. Apaf1-GFP without QVD: Median: 0%, MAD: 0%, N=167 cells examined over 2 independent experiments. At least 21 cells were imaged per condition and experiment. **e)** Time elapsed between the first detection of the fluorescent signal of Apaf1-ΔWD40-GFP and foci formation, in HeLa cells in absence or presence of QVD. Each point represents an individual cell. Each colour represents an experiment[1]. Medians of each experiment are indicated by large points. Black lines indicate medians with 95% confidence interval of all data points combined. Without QVD: Median: 45 min, MAD: 15 min, N=248 cells examined over 5 independent experiments. With QVD: Median: 45 min, MAD: 15 min, N=313 cells examined over 6 independent experiments. P-value=0.2789 (two-tailed Mann-Whitney test). **f)** Percentages of HeLa cells transiently transfected with Apaf1-ΔWD40-GFP, in presence or absence of QVD, showing Apaf1 foci disassembly, among all cells showing foci formation. Lines correspond to medians with 95% confidence interval.

Without QVD: Median: 92%, MAD: 1%, N=273 cells examined over 5 independent experiments. With QVD: Median: 80%, MAD: 8% N=340 cells examined over 6 independent experiments. At least 33 cells were imaged per condition and experiment. **g)** Lifetimes of Apaf1-ΔWD40-GFP foci in HeLa cells treated with QVD. Each point represents an individual cell. Each colour represents an experiment. Medians of each experiment are indicated by large points. Black lines indicate medians with 95% confidence interval of all data points combined. Median: 1 h 15 min, MAD: 30 min, N=232 cells examined over 6 experiments. P-value=0.0002 (*** two-tailed Mann-Whitney test) compared to the lifetimes of Apaf1-ΔWD40-GFP foci in HeLa cells not treated with QVD, shown in Fig. 5g. **h)** Western blots showing PARP and caspase-9 cleavage in HeLa WT cells, or HeLa cells transiently expressing Apaf1-GFP or Apaf1-ΔWD40-GFP, in presence or absence of ABT-737 and QVD. The two blots are from one single membrane cut into two; the upper part was incubated with an anti-PARP antibody, the lower part with an anti-caspase-9 antibody. Representative of 4 experiments with similar results. **i)** Western blot showing knockout of caspase-9 in HCT116 cells, detected using an anti-caspase-9 antibody. Representative of 2 experiments with similar results. **j)** Percentage of HeLa caspase-9KO cells transfected with Apaf1-SNAP in which Apaf1 foci also contained the caspase-9 construct with which the cells were co-transfected, as shown in Fig. 5j and in panel k here. Note that only images in which the field of view contained at least one cell with foci were acquired and analysed, thus the percentages are an overestimate compared to all other experiments. 3xFLAG-caspase-9 median: 5%, MAD: 2%, N=477 cells examined over 3 independent experiments. mCherry-caspase-9 median: 8%, MAD: 1%, N=424 cells examined over 2 independent experiments. At least 145 Apaf1-SNAP transfected cells were analysed per experiment and condition. We estimated that of the Apaf1-SNAP transfected cells, 67% were also transfected with caspase-9, based on the mCherry and SNAP647 signals. **k)** Fluorescence microscopy of fixed ABT-737/QVD treated HeLa caspase-9KO cells co-expressing Apaf1-SNAP, labelled with SNAP-Cell 647-SiR (green), and mCherry-caspase-9 (magenta). White square indicates area shown as close-ups (from left to right: Apaf1-SNAP; mCherry-caspase-9; merge). **l)** Immunofluorescence of ABT-737/QVD treated HeLa WT cells co-expressing Apaf1-SNAP and 3xFLAG-caspase-9, labelled with an anti-FLAG antibody (green) and SNAP-Cell 647-SiR (magenta). White square indicates area shown as close-ups (from left to right: FLAG antibody; Apaf1-SNAP; merge). Images in k and l are representative of two experiments each. Scale bars in c, k and l: 10 µm in large images, 3 µm in close-ups.

Supplementary Table 1: Oligonucleotide primers

Primer sequence	Primer use
GGGGACAAGTTTGTACAAAAAAGCAGGC TTCATGGATGCAAAAGCTCGAAATT	Fw primer to amplify Apaf1-XL cDNA for pCI-mEGFP-Apaf1 plasmid.
GGGGACCACTTTGTACAAGAAAGCTGGG TCTTATTCTAAAGTCTGTAAAATA	Rv primer to amplify Apaf1-XL cDNA for pCI-mEGFP-Apaf1 plasmid.
AAAAAGCTAGCGCCACCATGGATGCAAAA GCTCGA	Fw primer to amplify Apaf1-XL and Apaf1-XL(1-559aa) cDNA for pCI-Apaf1-mEGFP and for pCI-Apaf1-ΔWD40-mEGFP plasmids.
AAAAAGGTACCGAACTCGCCGCTGCCGG CGGCGCTGCCGGCGCTGCCTTCTAAAGT CTGTAAAATATATAAAATACCAAGATTATC	Rv primer to amplify Apaf1-XL cDNA for pCI-Apaf1-mEGFP plasmid.
GGGGACAAGTTTGTACAAAAAAGCAGGC TTAGCCACCATGGATGCAAAAGCT	Fw primer to amplify Apaf1-mEGFP for pLenti6/V5-EXPR-Apaf1-mEGFP plasmid.
GGGGACCACTTTGTACAAGAAAGCTGGG TTCTACTTGTACAGCTCGTCCATGC	Rv primer to amplify Apaf1-mEGFP for pLenti6/V5-EXPR-Apaf1-mEGFP plasmid.
AAAAAAGGTACCGAACTCGCCGCTGCCG GCGGCGCTGCCGGCGCTGCCTGGCTGT CGTCCAAGAAGGTGT	Rv primer to amplify Apaf1-XL(1-559aa) for pCI-Apaf1-ΔWD40-mEGFP plasmid.
TAAAAAAGTGAAGATTTGGAATTCTATGA CTGGGGAACTAGTACACA	Fw primer to amplify fragment-1 of Apaf1 for pCI-Apaf1-dead-mEGFP plasmid.
ATCAGGAGAAAACATCACACCATGAACAT CACTTAAATGTCCTCTGCAAT	Rv primer to amplify fragment-1 of Apaf1 for pCI-Apaf1-dead-mEGFP plasmid. Contains Trp884Asp mutation.
TGGTGTGATGTTTTCTCCTGATGGATCAT CATTTTTGACATCTTCTGA	Fw primer to amplify fragment-2 of Apaf1 for pCI-Apaf1-dead-mEGFP plasmid.
TTTTGCCATCTGGAGAAAAGCAAAGGTCA GTCACATCGCCTCCATGGGTA	Rv primer to amplify fragment-2 of Apaf1 for pCI-Apaf1-dead-mEGFP plasmid. Contains Trp1179Asp mutation.
TTTGCTTTTCTCCAGATGGCAAAATGCTTA TCTCTGCTGGAGGATATATT	Fw primer to amplify fragment-3 of Apaf1 for pCI-Apaf1-dead-mEGFP plasmid.
AACAGCTCCTCGCCCTTGCTCACCGGGG TACCGAACT	Rv primer to amplify fragment-3 of Apaf1 for pCI-Apaf1-dead-mEGFP plasmid.

GATCGATATCGAATTCACCGGTA	Fw primer to amplify SNAP-tag for pCI-Apaf1-SNAP-tag plasmid.
CTGGATCAGTTATCTATGCGCGGCCGCCCGGGT	Rv primer to amplify SNAP-tag for pCI-Apaf1-SNAP-tag plasmid.
TAATACGACTCACTATAGGCTAGCTAGACCATGGTGAGCAAGGGCGAGGAGGAT	Fw primer to amplify mCherry for pCI-N-terminal-mCherry plasmid.
AACTTGTGATGGGTCGACTCGAGAGCTTGTACAGCTCGTCCATGCCGCCGGT	Rv primer to amplify mCherry for pCI-N-terminal-mCherry plasmid.
TCAATGTATCTTATCATGTCTGCTCGAAGCGGCCGCCCATCACCACTTTGTACAAGAAAG	Fw primer to amplify caspase-9 for pCI-mCherry-caspase-9 plasmid.
TCCTCCTCGCCCTTGCTCACCATGGTCTAGCTAGCCTATAGTGAGTCGTATTAAGTACTC	Rv primer to amplify caspase-9 for pCI-mCherry-caspase-9 plasmid.
CTTTCTTGTACAAAGTGGTGATGGGCGGCCGCTTCGAGCAGACATGATAAGATACATTGA	Fw primer to amplify pCI-N-terminal-mCherry backbone for pCI-mCherry-caspase-9 plasmid.
GCTTTTTTGTACAAACTTGTGATGGGTCGACTCGAGAGCTTGTACAGCTCGTCCATGCCG	Rv primer to amplify pCI-N-terminal-mCherry backbone for pCI-mCherry-caspase-9 plasmid.
TCTGCTCGAAGCGGCC	Fw primer to amplify 3x-FLAG-caspase-9 for pCI-3x-FLAG-caspase-9 plasmid.
TACTTATATACTATAGGCTAGCGCCACC	Rv primer to amplify 3x-FLAG-caspase-9 for pCI-3x-FLAG-caspase-9 plasmid.
TCCCCCTGGGAAACAACCTTCT	Apaf1 sequencing primer.
TGTTTCCCAGGGGGAGTGCAT	Apaf1 sequencing primer.
AGACATCAAAGATTATTACACAGA	Apaf1 sequencing primer.
AGCTAAGCTGCAGGCCAAGCA	Apaf1 sequencing primer.
ATTAATGTGAAACAGTTCTTCCTA	Apaf1 sequencing primer.
ATTGAGATTTTAGAACTTGTAAACA	Apaf1 sequencing primer.
AGATGGCAAAATGCTTATCTCT	Apaf1 sequencing primer.
CGGCGCGGGTCTTGTAGTTG	Apaf1 sequencing primer.
ACAAATAAAGCATTTTTTTCACT	Apaf1 sequencing primer.
TGTCCTGGCCTGTGTCCTCTAA	caspase-9 sequencing primer.
AGTTGCGGCGTCGCTTCTCCT	caspase-9 sequencing primer.

TGAGACCCTGGACGACATCTTTGA	caspase-9 sequencing primer.
TTCAGGCCCCATATGATCGA	caspase-9 sequencing primer.
ATATATTAAGTGGTGGAACGTT	Apaf1end-Snap-tag sequencing primer.
CACCGCGCAGCAGTCCAGAGCACCG	Fw primer for caspase-9KO sgRNA (CGCAGCAGTCCAGAGCACCG).
AAACCGGTGCTCTGGACTGCTGCGC	Rv primer for caspase-9KO sgRNA (CGCAGCAGTCCAGAGCACCG).
GUCCUUAGGAUGCAAAUGA	siRNA for Apaf1 knockdown experiments.

REFERENCES:

1. Lord SJ, Velle KB, Mullins RD, Fritz-Laylin LK. SuperPlots: Communicating reproducibility and variability in cell biology. *J Cell Biol* **219**, (2020).

2. Kukulski W, Schorb M, Welsch S, Picco A, Kaksonen M, Briggs JA. Correlated fluorescence and 3D electron microscopy with high sensitivity and spatial precision. *J Cell Biol* **192**, 111-119 (2011).

3. Ader NR*, et al.* Molecular and topological reorganizations in mitochondrial architecture interplay during Bax-mediated steps of apoptosis. *eLife* **8**, e40712 (2019).

4. Ganeva I*, et al.* The architecture of Cidec-mediated interfaces between lipid droplets. *Cell Rep* **42**, 112107 (2023).

5. Bieber A*, et al.* In situ structural analysis reveals membrane shape transitions during autophagosome formation. *Proceedings of the National Academy of Sciences of the United States of America* **119**, e2209823119 (2022).

6. Wozny MR*, et al.* In situ architecture of the ER-mitochondria encounter structure. *Nature* **618**, 188-192 (2023).

7. Yang J*, et al.* Precise 3D Localization by Integrated Fluorescence Microscopy (iFLM) for Cryo-FIB-milling and In-situ Cryo-ET. *Microsc Microanal* **29**, 1055-1057 (2023).
