# Supplementary material for: Large transient assemblies of Apaf1 constitute the apoptosome in cells
Source: Nat Commun. 2025 Oct 24;16:9429. doi: 10.1038/s41467-025-64478-9 (PMC12552632; doi:10.1038/s41467-025-64478-9)
Supplement: Supplementary file 2 — Description of Additional Supplementary Information [file 41467_2025_64478_MOESM2_ESM.pdf]

Description of Additional Supplementary Files

File Name: Supplementary Movie 1
Description: *Live fluorescence imaging of HeLa cells stably expressing Apaf1- GFP, treated with ABT-737.* Movie showing Apaf1-GFP (green) foci formation followed by cell shrinkage and death. Mitochondria were stained with MitoTracker DeepRed (magenta). Image acquisition time (h:min) after ABT-737 treatment is indicated at the top left. Imaging frame rate: 15 minutes. Scale bar: 10 µm

File Name: Supplementary Movie 2
Description: *Live fluorescence imaging of a HeLa cell stably expressing Apaf1- GFP, treated with ABT-737.* Movie showing Apaf1-GFP (green) foci formation and disassembly in an apoptotic cell. Mitochondria were stained with MitoTracker DeepRed (magenta). Image acquisition time (h:min) after ABT-737 treatment is indicated at the top left. Imaging frame rate: 15 minutes. Scale bar: 10 µm.

File Name: Supplementary Movie 3
Description: *Live fluorescence imaging of a HeLa cell stably expressing Apaf1- GFP, treated with ABT-737 and QVD.* Movie showing Apaf1-GFP (green) foci formation and disassembly in apoptotic cells. Mitochondria were stained with MitoTracker DeepRed (magenta). Image acquisition time (h:min) after ABT-737 and QVD treatment is indicated at the top left. Imaging frame rate: 15 minutes. Scale bar: 10 µm.

File Name: Supplementary Movie 4
Description: *Live fluorescence imaging of a HeLa cell stably expressing Apaf1- GFP, treated with ABT-737 and QVD.* Movie showing two consecutive events of Apaf1-GFP (green) foci formation in an apoptotic cell. Mitochondria were stained with MitoTracker DeepRed (magenta). Image acquisition time (h:min) after ABT-737 treatment is indicated at the top left. Imaging frame rate: 15 minutes. Scale bar: 10 µm.

File Name: Supplementary Movie 5
Description: *Electron tomogram of Apaf1-GFP in resin-embedded cell.* Movie through virtual slices of the electron tomogram shown in Figure 3B, obtained by CLEM on resinembedded HeLa cells expressing Apaf1-GFP, treated with ABT-737 and QVD. Scale bar: 200 nm.

File Name: Supplementary Movie 6
Description: *Electron tomogram of Apaf1-GFP in resin-embedded cell.* Movie through virtual slices of the electron tomogram shown in Figure 3D, obtained by CLEM on resinembedded HeLa cells expressing Apaf1-GFP, treated with ABT-737 and QVD. Scale bar: 200 nm.

File Name: Supplementary Movie 7
Description: *Live fluorescence imaging of a HeLa cell transiently expressing Apaf1-SNAP, treated with ABT-737 and QVD.* Movie showing Apaf1-SNAP647 (magenta) foci formation and disassembly in apoptotic cells. Mitochondria were stained with MitoSpy green (green). Image acquisition time (h:min) after ABT-737 and QVD treatment is indicated at the top left. Imaging frame rate: 15 minutes. Scale bar: 10 µm.

File Name: Supplementary Movie 8
Description: *Cryo-electron tomogram of Apaf1-GFP in vitrified, cryo-FIB-milled cell.* Movie through virtual slices of the cryo-electron tomogram shown in Figure 3G, obtained by preFIB milling cryo-CLEM of HeLa cells expressing Apaf1-GFP, treated with ABT-737 and QVD. Scale bar: 100 nm.

File Name: Supplementary Movie 9
Description: *Cryo-electron tomogram of Apaf1-SNAP647 in vitrified, cryo-FIBmilled cell.* Movie through virtual slices of the cryo-electron tomogram shown in Figure 3L, obtained by pre- and post-FIB milling cryo-CLEM of HeLa cells expressing Apaf1-SNAP-tag labelled with SNAP-Cell 647-SiR, treated with ABT-737 and QVD. Scale bar: 100 nm.

File Name: Supplementary Movie 10
Description: *Live fluorescence imaging of a HeLa cells stably expressing Apaf1- GFP, microinjected with cyt c.* Movie showing Apaf1-GFP (green) foci formation and dynamics upon microinjection of cyt c and rhodamine dextran (magenta). Imaging time (h:min) after the start of the microinjection session is indicated at the top left. Imaging frame rate: 10 minutes. Scale bar: 10 µm.

File Name: Supplementary Movie 11
Description: *Live fluorescence imaging of a HeLa cells expressing Apaf1-DWD40- GFP.* Movie showing Apaf1-DWD40-GFP (green) foci formation and disassembly upon expression in cells. Imaging time (h:min) after the transfection is indicated at the top left. Imaging frame rate: 15 minutes. Scale bar: 10 µm.
