# Supplementary material for: Large transient assemblies of Apaf1 constitute the apoptosome in cells
Source: Nat Commun. 2025 Oct 24;16:9429. doi: 10.1038/s41467-025-64478-9 (PMC12552632; doi:10.1038/s41467-025-64478-9)
Supplement: Supplementary file 14 — Reporting Summary [file 41467_2025_64478_MOESM14_ESM.pdf]

nature portfolio

Corresponding author(s): Wanda Kukulski

Last updated by author(s): 2025/09/11

# Reporting Summary

Nature Portfolio wishes to improve the reproducibility of the work that we publish. This form provides structure for consistency and transparency in reporting. For further information on Nature Portfolio policies, see our Editorial Policies and the Editorial Policy Checklist.

## Statistics

For all statistical analyses, confirm that the following items are present in the figure legend, table legend, main text, or Methods section.

| n/a | Confirmed | |
|---|---|---|
| ☐ | ☒ | The exact sample size (*n*) for each experimental group/condition, given as a discrete number and unit of measurement |
| ☐ | ☒ | A statement on whether measurements were taken from distinct samples or whether the same sample was measured repeatedly |
| ☐ | ☒ | The statistical test(s) used AND whether they are one- or two-sided<br>*Only common tests should be described solely by name; describe more complex techniques in the Methods section.* |
| ☐ | ☒ | A description of all covariates tested |
| ☒ | ☐ | A description of any assumptions or corrections, such as tests of normality and adjustment for multiple comparisons |
| ☐ | ☒ | A full description of the statistical parameters including central tendency (e.g. means) or other basic estimates (e.g. regression coefficient) AND variation (e.g. standard deviation) or associated estimates of uncertainty (e.g. confidence intervals) |
| ☐ | ☒ | For null hypothesis testing, the test statistic (e.g. *F*, *t*, *r*) with confidence intervals, effect sizes, degrees of freedom and *P* value noted<br>*Give P values as exact values whenever suitable.* |
| ☒ | ☐ | For Bayesian analysis, information on the choice of priors and Markov chain Monte Carlo settings |
| ☒ | ☐ | For hierarchical and complex designs, identification of the appropriate level for tests and full reporting of outcomes |
| ☒ | ☐ | Estimates of effect sizes (e.g. Cohen's *d*, Pearson's *r*), indicating how they were calculated |

*Our web collection on statistics for biologists contains articles on many of the points above.*

## Software and code

Policy information about availability of computer code

| | |
|---|---|
| Data collection | Live fluorescence microscopy and immunofluorescence: ZEN imaging software (Zeiss), immunofluorescence and room-terperature CLEM: NIS Elements software, cryo-fluorescence microscopy: LAS X (Leica Microsystems) and iFLM software (Thermo Fisher Scientific), cryo-FIB milling: AutoTEM Cryo software (Thermo Fisher Scientific), room temperature ET: SerialEM (https://bio3d.colorado.edu/SerialEM/) cryo-ET: Tomography 5 (Thermo Fisher Scientific), |
| Data analysis | MATLAB (MathWorks), Prism (Graphpad), R/Rstudio (R Core Team), IMOD (https://bio3d.colorado.edu/imod/), UCSF ChimeraX, Fiji, ecCLEM / Icy, CellProfiler. |

For manuscripts utilizing custom algorithms or software that are central to the research but not yet described in published literature, software must be made available to editors and reviewers. We strongly encourage code deposition in a community repository (e.g. GitHub). See the Nature Portfolio guidelines for submitting code & software for further information.

## Data

Policy information about availability of data

All manuscripts must include a data availability statement. This statement should provide the following information, where applicable:

- Accession codes, unique identifiers, or web links for publicly available datasets
- A description of any restrictions on data availability
- For clinical datasets or third party data, please ensure that the statement adheres to our policy

Electron tomograms (Fig. 3) are deposited on EMDB with accession numbers EMD-54278, EMD-54279, EMD-54280 and EMD-54281. All other data are contained in

the manuscript or available on request from the authors.

## Research involving human participants, their data, or biological material

Policy information about studies with human participants or human data. See also policy information about sex, gender (identity/presentation), and sexual orientation and race, ethnicity and racism.

| | |
|---|---|
| Reporting on sex and gender | n/a |
| Reporting on race, ethnicity, or other socially relevant groupings | n/a |
| Population characteristics | n/a |
| Recruitment | n/a |
| Ethics oversight | n/a |

Note that full information on the approval of the study protocol must also be provided in the manuscript.

# Field-specific reporting

Please select the one below that is the best fit for your research. If you are not sure, read the appropriate sections before making your selection.

[x] Life sciences [ ] Behavioural & social sciences [ ] Ecological, evolutionary & environmental sciences

For a reference copy of the document with all sections, see nature.com/documents/nr-reporting-summary-flat.pdf

# Life sciences study design

All studies must disclose on these points even when the disclosure is negative.

| | |
|---|---|
| Sample size | Fluorescence live imaging experiments were performed as at least three independent experimental repeats except as indicated, using biological replicates. Within each experiment, the number of cells to be imaged was not pre-determined. For data acquisition by ET and cryo-ET, the maximum available samples were used. |
| Data exclusions | CLEM, ET and cryo-ET samples and data were assessed based on quality. Data were excluded if any of the following occurred: Sample was poorly vitrified, contaminated, or data acquisition problems impaired data quality.<br>For Live-imaging, all imaging session were use if the sample was good. When technical issues affected sample quality, for example poor transfection efficiency or a failed staining, samples were not used further. Within a data set, if the timing or type of event happening in a cell was poorly defined, the cell was excluded for analysis. |
| Replication | Live cell imaging , single microinjection and immunofluorescence experiments were repeated three times or more, as indicated. |
| Randomization | There were no experimental groups requiring randomization |
| Blinding | No separate treatment groups are included in the analysis |

# Reporting for specific materials, systems and methods

We require information from authors about some types of materials, experimental systems and methods used in many studies. Here, indicate whether each material, system or method listed is relevant to your study. If you are not sure if a list item applies to your research, read the appropriate section before selecting a response.

### Materials & experimental systems

| n/a | Involved in the study | |
|---|---|---|
| [ ] | [x] | Antibodies |
| [ ] | [x] | Eukaryotic cell lines |
| [x] | [ ] | Palaeontology and archaeology |
| [x] | [ ] | Animals and other organisms |
| [x] | [ ] | Clinical data |
| [x] | [ ] | Dual use research of concern |
| [x] | [ ] | Plants |

### Methods

| n/a | Involved in the study | |
|---|---|---|
| [x] | [ ] | ChIP-seq |
| [ ] | [x] | Flow cytometry |
| [x] | [ ] | MRI-based neuroimaging |

# Antibodies

| | |
|---|---|
| Antibodies used | For western blots: Primary: Rabbit anti-Apaf1 SY22-02 (Invitrogen, MA5-32082), mouse anti-beta-actin (Sigma, A5316), mouse anti-caspase-9, mouse anti-PARP antibody (BD Biosciences, 556362) Secondary: anti-rabbit-HRP (Invitrogen, 65-6120), anti-mouse-HRP (Dako, P0260).<br>For immunofluorescence: Primary: Rabbit anti-Apaf1 (Invitrogen, PA5-19893) , mouse IgG2a anti-Tom20 (Santa Cruz Biotechnology, Sc-17764), mouse anti-cytochrome c (BD Pharmingen, 556432), and rabbit anti-FLAG (DYDDDDK peptide) (Cell Signaling, 14793S). Secondary: Goat anti-rabbit Alexa-Fluor 488 (Invitrogen, A11034), donkey anti-rabbit Alexa-Fluor 488 (Invitrogen, A21206), goat anti-mouse Alexa-Fluor 647 (Invitrogen, A32728), goat anti-mouse IgG2a Alexa-Fluor 647 (Invitrogen, A21241), goat anti-rabbit Alexa-Fluor 568 (Invitrogen, A11011), and goat anti-mouse-IgG1 Alexa-Fluor 568 (Invitrogen, A21124). |
| Validation | For all primary antibodies, validation was either deduced from the manufacturer's website or from cited publications. For all primary antibodies, we further validated their efficiency by appropriate experimental controls. |

# Eukaryotic cell lines

Policy information about cell lines and Sex and Gender in Research

| | |
|---|---|
| Cell line source(s) | HeLa cells, HCT116 cells, U2OS cells |
| Authentication | none of the cell lines was authenticated |
| Mycoplasma contamination | All cell lines were tested regularly and were negative for mycoplasma contamination |
| Commonly misidentified lines (See ICLAC register) | n/a |

# Plants

| | |
|---|---|
| Seed stocks | n/a |
| Novel plant genotypes | n/a |
| Authentication | n/a |

# Flow Cytometry

## Plots

Confirm that:

☐ The axis labels state the marker and fluorochrome used (e.g. CD4-FITC).

☐ The axis scales are clearly visible. Include numbers along axes only for bottom left plot of group (a 'group' is an analysis of identical markers).

☐ All plots are contour plots with outliers or pseudocolor plots.

☐ A numerical value for number of cells or percentage (with statistics) is provided.

## Methodology

| | |
|---|---|
| Sample preparation | Flow cytometry was used as a supplementary control experiment to assess cell death stages; it is not a central method in the paper. Results are shown in Supplementary Figure 2c as a bar diagram from a single experiment; no scatter plots are shown, therefore the above tickboxes do not apply.<br>HeLa cells stably expressing Apaf1-GFP were used for flow-cytometry. The culture medium and washes were collected. Cells were scraped and collected with the medium. Cells were incubated with Atto633-Annexin V for at least 20 min on ice in the dark and then washed. Propidium iodide (Sigma, 81845) was added to a final concentration of 2 µg/mL. For controls (negative: unstained, positive: stained with Atto633-Annexin V and propidium iodide), HeLa cells stably expressing Apaf1-GFP were treated with 1µM staurosporine for 2 hours to obtain a 1:1 population of apoptotic to healthy cells. For calibration of the FACS machine, staurosporine-induced cells were stained with Atto633-Annexin V or Propidium iodide. |
| Instrument | FACSLyric flow cytometer (BD Biosciences) |
| Software | FlowJO software |
| Cell population abundance | n/a |

| Gating strategy | Identical preliminary FSC/SSC gate was set to exclude debris. Quadrants were then set using the positive control sample (1:1 mix of healthy and apoptotic cells) in the Atto633-Annexin V / PI dot plot to distinguish living from apoptotic cells. |
|---|---|

☐ Tick this box to confirm that a figure exemplifying the gating strategy is provided in the Supplementary Information.
